# Chemical Doping Engineering of Polarization and Topological Textures in van der Waals Ferroelectric CuInP_2_S_6_

**DOI:** 10.1002/advs.202523774

**Published:** 2026-01-05

**Authors:** Lei Gao, Wenhao Li, Fei Sun, Ziyi Zhou, Zhihao Shen, Jianwei Liang, He Jiang, Weiming Xiong, Weijin Chen, Xiaoyue Zhang, Yi Zhang, Xinzhi Liu, Yue Zheng

**Affiliations:** ^1^ Guangdong Provincial Key Laboratory of Magnetoelectric Physics and Devices Centrer for Physical Mechanics and Biophysics Institute of Neutron Science and Technology School of Physics Sun Yat‐sen University Guangzhou P. R. China; ^2^ State Key Laboratory of Optoelectronic Materials and Technologies Sun Yat‐sen University Guangzhou P. R. China; ^3^ Guangdong Provincial Key Laboratory of Magnetoelectric Physics and Devices Centrer for Physical Mechanics and Biophysics School of Physics Sun Yat‐sen University Guangzhou P. R. China; ^4^ School of Materials Sun Yat‐sen University Guangzhou P. R. China; ^5^ Center on Nanoenergy Research Guangxi Key Laboratory for the Relativistic Astrophysics School of Physical Science & Technology Guangxi University Nanning P. R. China

**Keywords:** CuInP_2_S_6_, flexoelectric effect, multiple polarization states, topological polar texture, vdW ferroelectrics

## Abstract

Precise modulation of ferroelectric properties in van der Waals (vdW) layered materials is crucial for multifunctional nanoelectronics. Here, we demonstrate that Li⁺ substitution at Cu sites (Cu_1‐x_Li_x_InP_2_S_6_, *x*≤ 0.1) concurrently enhances ferroelectric and ionic conductivities, and enriches the polarization configurations of CuInP_2_S_6_. With Li‐doping, benefiting from the strengthened Cu‐S interlayer bond and reduced interlayer spacing, the Curie temperature increases from 315 K (pristine) to 327 K (*x* = 0.1), and Cu⁺ ionic conductivity activation energy is lowered from ∼0.6 eV (pristine) to ∼0.4 eV (*x* = 0.1). Meanwhile, the high‐polarization state is stabilized and gives rise to the coexistence of low‐ and high‐polarization states (LP and HP), which lays a fertile ground for topological polar texture. Numerous polar bubbles and labyrinth domains with varied sizes and shapes are observed at the border between HP and LP phases. Interestingly, a strain gradient switches the HP to the LP state, establishing an intriguing flexoelectrical response and providing a mechanical pathway to tune and confine the polar texture. This work provides a feasible chemical strategy to modulate ferroelectric properties and polar configurations in vdW ferroelectrics, offering promising opportunities for advanced electronics, neuromorphic computing, and topotronics.

## Graphical abstract

Our work reports that Li⁺ doping in CuInP_2_S₆ induces coexistence of high‐ and low‐polarization states, which fosters a rich spectrum of topological polar textures, such as bubbles and labyrinth domains. An unconventional flexoelectric effect is also discovered, where strain gradients can mechanically manipulate these polarization states and their topological features.

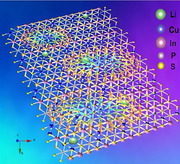

## 1 Introduction

The family of layered thio‐ phosphates, such as CuInP_2_S_6_, CuCrP_2_S_6_, CuVP_2_S_6_, has garnered significant attention due to their unique polar properties as 2D van der Waals materials [1, 2, 3, 4]. These compounds host a rich spectrum of ferroic orders, including ferro‐ and antiferroelectric [5], ferro‐ and antiferromagnetism [6], and extending to more exotic states such as polar bubbles [7] and altermagnetism [8]. Among them, CuInP_2_S_6_ (CIPS) stands out as a representative van der Waals (vdW) material which crystallizes in a monoclinic structure (space group *Cc*) below 315 K [9]. The ordered arrangement of Cu⁺ and In^3^⁺ in a single layer induces out‐of‐plane and in‐plane electrical polarizations, establishing CIPS as an intrinsic uniaxial multi‐well ferroelectric (FE) system [10, 11, 12]. Notably, its rich switchable polarization states, coupled with pronounced copper‐ion conductivity [13], confer extraordinary tunability under diverse external stimuli, e.g., electric field [14], mechanical stress [15], and thermal gradient [10]. The synergy between reconfigurable dipole arrangements and ion migration enables diverse device applications in memory, logic, and neuromorphic computing [16, 17, 18, 19, 20, 21, 22].

Of all the fascinating properties, the multiple stable positions of Cu^+^ with appropriate energy barriers play a dominant role, giving rise to both the unique quadruple polar states (low‐polarization (LP) and high‐polarization (HP)) and ferroionic behavior. The LP state corresponds to a polarization of ∼4 µC/cm^2^, while the HP state has a polarization of ∼10 µC/cm^2^ [23]. The LP state features Cu⁺ positioned at the center of S_3_ triangle on one side of S_6_ octahedron with a *C*_3v_ local symmetry. In contrast, the HP state locates above the LP position within the van der Waals gap, forming a distorted *T_d_* symmetric CuS_4_ tetrahedron with a sulfur atom from the neighboring layer [24]. The energy barrier between LP and HP sites has been found to be tiny and can be modulated by the electric field and stress. Particularly, the LP state shows a rare negative and high amplitude piezoelectric response, while the HP state has a normal positive but smaller piezo coefficient.

The multiple polar states strongly correlate with the chemical interaction within the gap, which fundamentally represents a competition between the energies of *C_3v_* and *T_d_* positions. Density functional theory (DFT) calculations have revealed that the *T_d_* position is stabilized by a weak Cu─S bond interaction across the gap [24]. In this interaction, the Cu 3*d* orbital plays a key role via *3d‐3p* hybridization, although Cu^+^ 3*d* orbital is fully filled. With a reduced gap spacing, the Cu─S bond has been proven to be strengthened, and HP state is stabilized [24]. This has been experimentally confirmed under hydrostatic pressure [25]. The rich polarization states also enable the versatile switching process by electric field, including from LP to HP within a single layer, or from + HP site to ‐HP site across the layer, giving rise to an intriguing reversed polarization with respect to the field [13, 26]. Furthermore, it has been found that the coexistence of LP and HP states enables high‐density polarization bubbles, providing a fertile ground for the topological polarization structures [27]. Concurrently, the low energy barrier enables significant Cu⁺ ion migration, making CIPS a promising ferroionic material for neuromorphic applications.

Chemical doping represents a straightforward approach to tuning material properties. However, its application in modifying the ferroelectricity of CIPS has largely yielded suppressive effects. Most reported substitutions (e.g., Ag⁺, Cr^3^⁺, Se^2^^−^) suppress ferroelectricity, lower *T*_c_, or induce dipolar glass behavior [6, 28, 29], which is detrimental for CIPS as a room temperature ferroelectric or ferroionic material. In contrast, the stress has proven to be an effective approach to tuning the Curie temperature and polarization magnitude. A small pressure of a few hundred MPa can enhance the *T_c_* by tens of kelvins [30], and a hydrostatic pressure of 0.26 GPa can stabilize the HP state as ground state, while ∼2.68 GPa pressure can fully suppress the FE property [25]. In particular, pressure aging treatment has been shown to yield CIPS with a *T_c_* as high as ∼580 K [31]. So far, the HP state or enhanced Curie temperature has been observed in electrically poled samples, strained samples under pressure [31], vertical and in‐plane heterostructure systems [32, 33, 34, 35]. This suggests that modulating the interlayer Cu─S interaction is key to controlling ferroelectricity. However, a chemical strategy that simultaneously enhances both ferroelectric and ionic properties under ambient conditions remains elusive.

In this work, we demonstrate that minimal Li⁺ doping (≤10%) on the Cu site provides a powerful chemical strategy to concurrently elevate the Curie temperature and stabilize the coexistence of LP and HP states. Although it had been predicted that Li fully substituting for Cu would destroy the FE, our findings validate that low‐level doping maintains the ferroelectric order and enriches the polarization configuration, therefore establishing a feasible chemical pathway to co‐optimize ferroelectric and ionic properties in CIPS.

## 2 Results and Discussions

### 2.1 Sample Preparation and Characterizations

**FIGURE 1:**
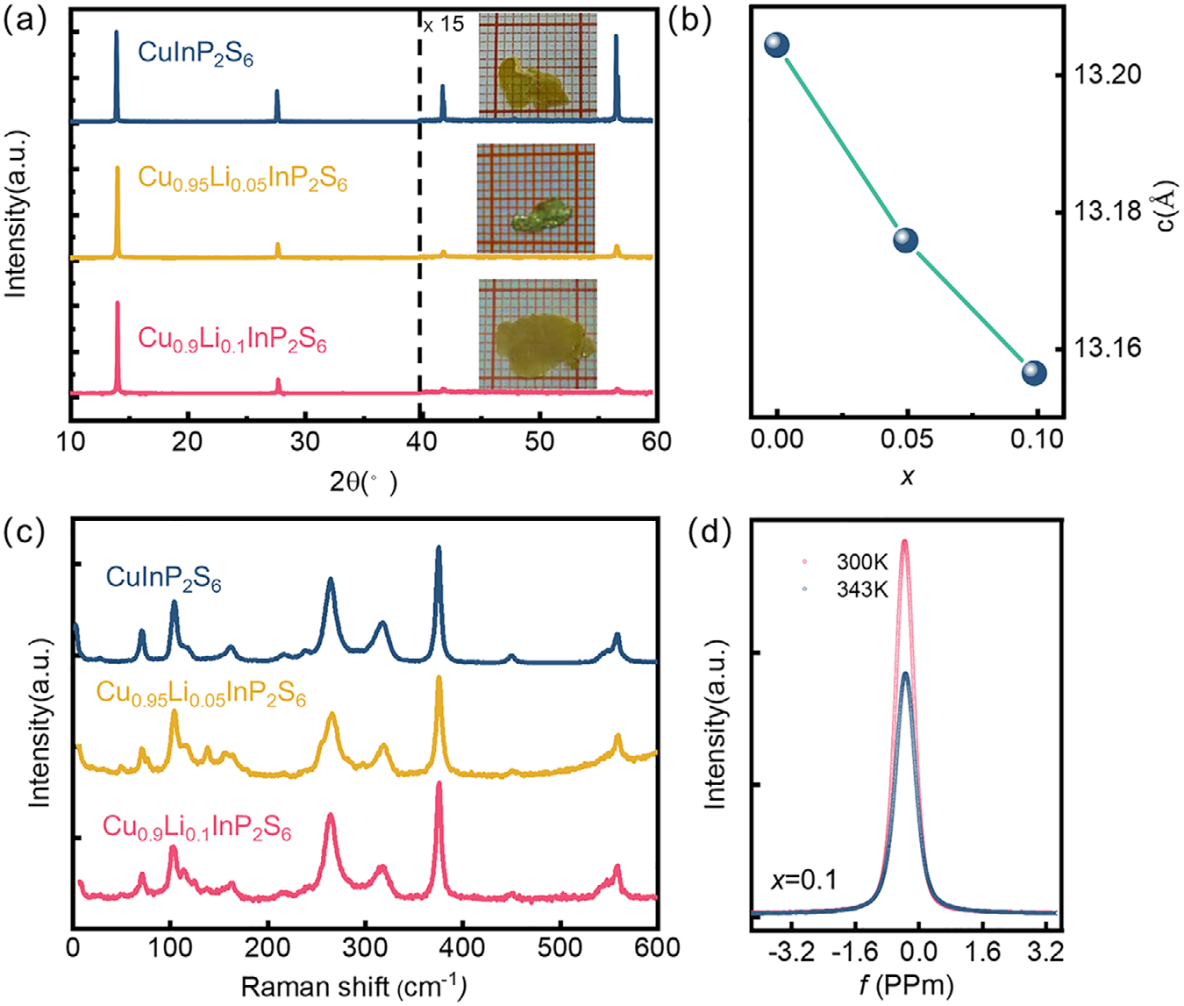
Structure characterization of Cu_1‐x_Li_x_InP_2_S_6_. (a) X‐ray diffraction of Cu_1‐x_Li_x_InP_2_S_6_ (*x* = 0, 0.05, 0.1) single crystals; (b) *c*‐axis parameter as a function of chemical composition; (c) Raman spectrum of Cu_1‐x_Li_x_InP_2_S_6_ at room temperature; (d) NMR spectra of Li at 300 and 343 K.

**FIGURE 2:**
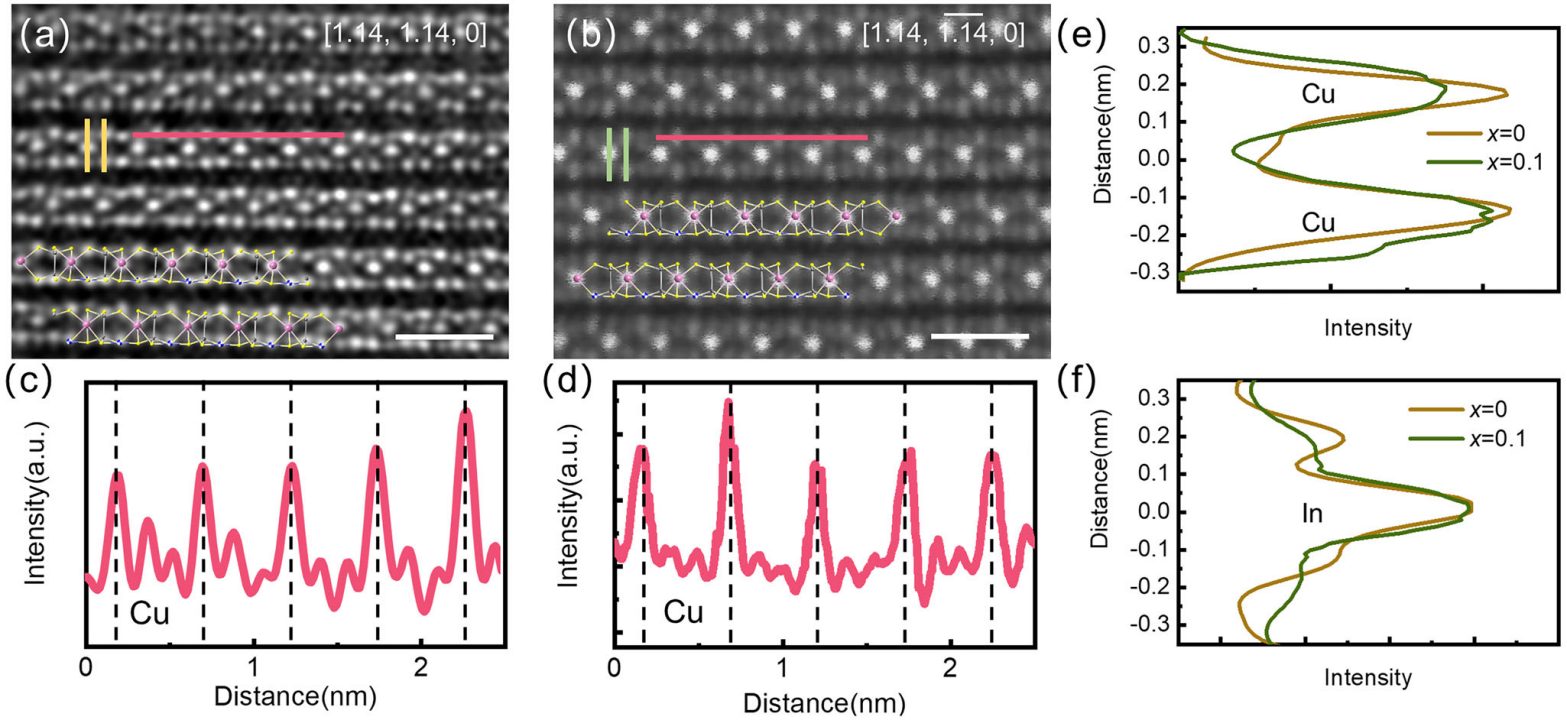
Atomic‐level imaging for CIPS and *x* = 0.1 Li‐doped CIPS. STEM picture of the (a)CIPS and (b) *x* = 0.1 Li‐doped CIPS. The FE *Cc* structure model is also mapped into the tomography. The scale bar is 1 nm. The row of atoms corresponding to the intensities of (c) CIPS and (d) *x* = 0.1 Li‐doped CIPS; The columns of (e) Cu and (f) In corresponding intensities.

High‐quality Cu_1‐x_Li_x_InP_2_S_6_ single crystals (*x* = 0, 0.05, 0.1) were grown by the chemical vapor transport (CVT) method (details are given in methods), some pictures of crystals are shown in Figure 1a. The sample quality was verified by X‐ray diffraction (XRD). A group of (00*L*) reflections along the *c*‐axis direction were observed, confirming the single‐crystal nature. The Bragg reflections gradually shift to a higher angle with Li‐doping, indicating a decreased interlayer spacing. The *c* parameter decreases from 13.204 Å for *x* = 0 to 13.175 Å and 13.156 Å for *x* = 0.05 and 0.1, respectively (Figure 1b). Rietveld refinement of Cu_0.9_Li_0.1_InP_2_S_6_ XRD powder pattern using the FE *Cc* structure confirmed successful Li incorporation without secondary phase (Figure S1). The *a* and *b* parameters slightly increase from 6.102 Å and 10.574 Å, to 6.115 Å and 10.587 Å for *x* = 0 to 0.1, respectively. The slight contraction along the *c*‐axis proves the doping is successful. Elemental mapping via energy‐dispersive X‐ray spectroscopy (EDS) demonstrated that elemental distributions match nominal stoichiometry for Cu, In, P, and S (Figure S2). While conventional EDS cannot detect Li due to its low X‐ray emission energy, a significant single NMR peak of the ^7^Li also suggests that successful doping was conducted. NMR spectroscopy also provided crucial insight into the chemical environment of Li, the single narrow peak proves a single site is occupied. The NMR pattern below and above the phase transition, collected at 300 and 343 K (Figure 1d) exhibits no change in chemical shift, indicating Li doesn't change its position. Furthermore, the high‐angle annular dark‐field scanning transmission electron microscopy (HAADF‐STEM) images for both CIPS and *x* = 0.1 Li‐doped CIPS show a good crystal quality in Figure 2a,b. The image directly resolves the atomic columns of In, Cu, P, and S, where the brightness intensity scales with the atomic number. The perfectly aligned atomic arrangement exhibits the crystalline structure consistent with the theoretical model with *Cc* SGP from the ICSD database, as also shown in Figure 2a,b.

To further characterize the influence of Li‐dopant on the structure and lattice dynamics, lattice vibration modes were analyzed via Raman spectroscopy. Room‐temperature Raman spectroscopy on samples with different doping ratios using a 532 nm laser is displayed in Figure 1c. For *x* from 0 to 0.1, the Raman spectra at room temperature reveal that all samples adopt the same structure as FE‐phase CIPS [36]. In particular, the peak near 320 cm^−1^ is closely correlated with the Cu ions occupation, therefore serves as an indication of FE‐PE transition [36, 37, 38]. All patterns with *x* = 0‐0.1 show a strong peak around 320 cm^−1^. For *x* = 0.1, the peak width is slightly broader than that of CIPS, indicating a partial disorder or distortion caused by Li‐doping.

**FIGURE 3:**
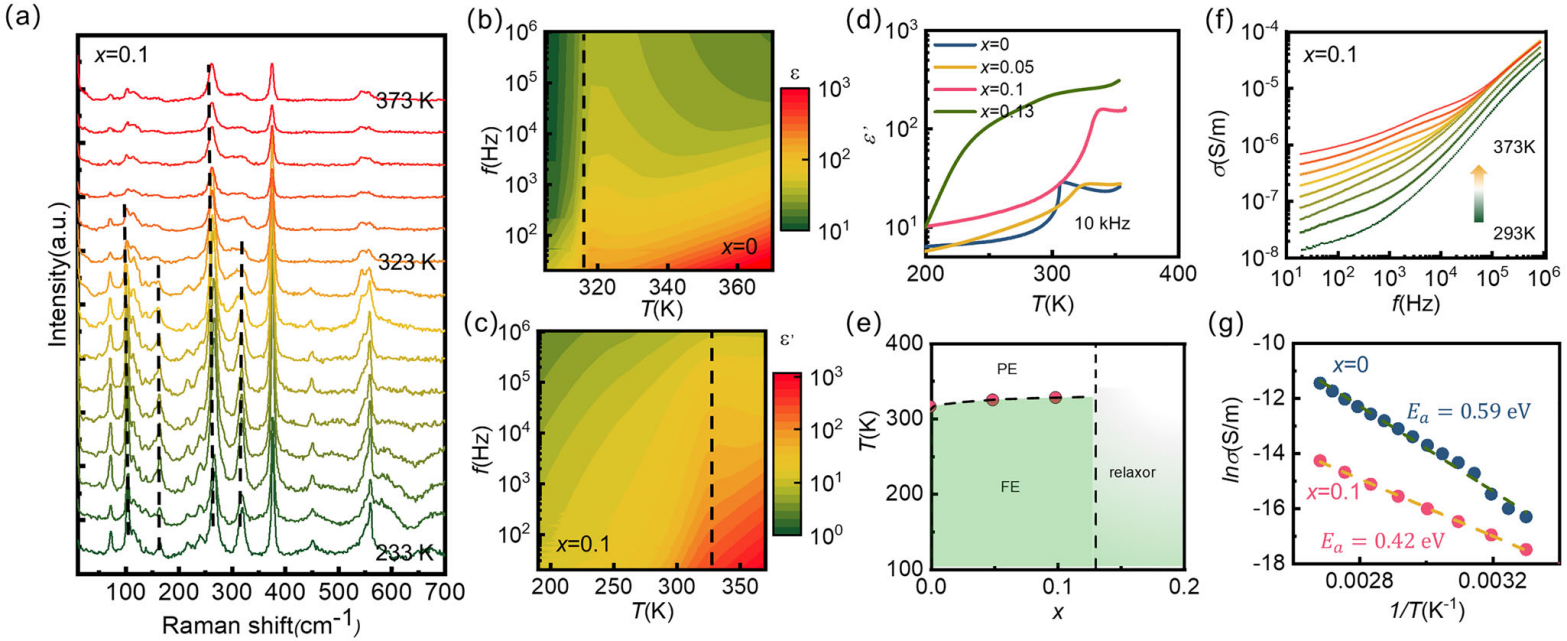
The Raman spectrum and dielectric of Cu_1‐x_Li_x_InP_2_S_6_ as a function of temperature. (a) Raman spectroscopy of mechanically exfoliated *x* = 0.1 flake with different temperatures; (b) Contour plots of the dielectric real part of permittivity ε′ over the range of 300−370 K for CuInP_2_S_6_; (c) Contour plots of ε′ over the range of 180−370 K for Cu_0.9_Li_0.1_InP_2_S_6_. (d) The temperature dependence of ε′ of Cu_1‐x_Li_x_InP_2_S_6_ at 10 kHz;(e) summarized phase diagram of the Cu_1‐x_Li_x_InP_2_S_6_ system (f) The frequency dependence of σ for Cu_0.9_Li_0.1_InP_2_S_6_ at different temperatures; (g) Arrhenius plot for CIPS and Cu_0.9_Li_0.1_InP_2_S_6_ and fitting to the temperature‐dependent conductivity σ.

Subsequently, we further discuss the temperature‐dependent Raman spectra during the FE‐PE phase transition. By examining the temperature‐dependent evolution of Raman spectra for *x* = 0.1 sample in Figure 3a, Li doping's influence on FE‐PE phase transition is disclosed. Above 327 K, the intensities of several peaks weaken or disappear, suggesting the phase transition. By comparing the peak positions and intensities, it was found that these peaks exhibit discontinuous behavior around the phase transition. Especially, the vanishment of the peak at 320 cm^−1^ indicates Cu^+^ becomes disordered in the S_6_ framework above the transition temperature.

To systematically investigate the influence of Li doping on the ferroelectric properties of CIPS, the dielectric spectra for different doping levels at variable temperatures were measured using an impedance analyzer across a frequency range from 20 Hz to 10^6^ Hz at different temperatures. Figure 3b,c present the real part of the dielectric constant (*ε'*) for CIPS and *x* = 0.1 Li doped CIPS, respectively. Both samples exhibit characteristic FE‐PE transitions, with *T_c_* increasing significantly upon Li doping. While CIPS shows a transition at 315 K, which is consistent with previous reports [39, 40], *x* = 0.1 sample demonstrates an elevated transition temperature of ∼327 K, consistent with the Raman spectrum. This Li doping‐induced transition temperature variation shows excellent reproducibility, as evidenced by measurements of multiple samples (Figure S4). At 10 kHz, the temperature‐dependent dielectric investigations of the three crystals show the transition temperature of 315 K (*x* = 0), 324 K (*x* = 0.05), and 327 K (*x* = 0.1) as shown in Figure 3d. Furthermore, a dielectric pattern of *x* = 0.13 sample is also shown, which illustrates that the FE is destroyed while a relaxor behavior is evidenced at about 150–300 K. In Figure 3e, a phase diagram of the Li‐doped CIPS system is summarized by combining the dielectric and Raman data.

In addition to the FE property, the dielectric spectrum also revealed a significant influence on ionic conductivity caused by Li^+^ doping. Figure S5 illustrates the frequency‐dependent evolution of the imaginary part ε′′ of the dielectric constant at various temperatures. At low frequencies, the conductivity phenomena dominate in the dielectric spectra. Figure 3d displays the conductivity σ = 2π*f*ε_0_ε′′as a function of frequency at various temperatures for *x* = 0.1 Li‐doped CIPS, where σ denotes the electric conductivity, *f* is frequency, and ε_0_ is vacuum permittivity [40]. σ can be divided as σ = σ_*DC*_ + *A*ω^*s*^, where σ_*DC*_ is the DC conductivity and *A*ω^*s*^ is the AC conductivity. At low frequency, σ ≈ σ_*DC*_. Through fitting of the Arrhenius equation $\sigma =\sigma_{0}\exp\left(-\Delta E/kT\right)$, where Δ*E* is activation energy, *k* is Boltzmann constant and *T* is temperature, the activation energy Δ*E* is obtained. For CIPS, Δ*E* is found to be 0.59 eV (Figure 3g), similar to the previous reports [40], while *x* = 0.1 sample exhibited a reduced Δ*E* of 0.42 eV. The variation in the activation energy reveals that Li doping enhances ion migration under electric fields.

### 2.2 PFM Measurements and Piezoelectric Response

**FIGURE 4:**
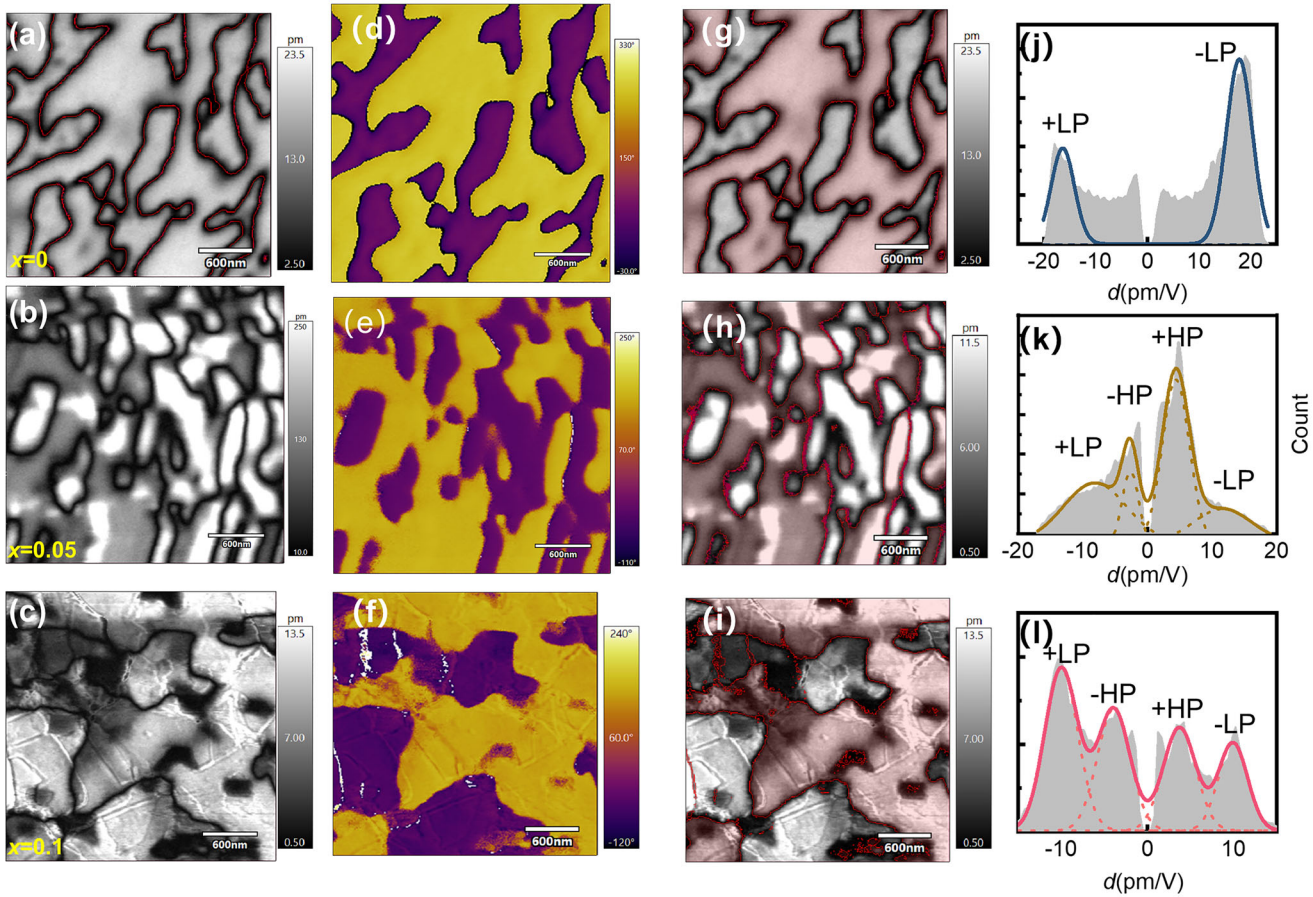
PFM spectrum for CIPS (*x* = 0) and Li‐doped CIPS with *x* = 0.05, 0.1. (a–c) the Out‐of‐plane PFM amplitude of samples with *x* = 0, 0.05 and 0.1. (d–f) the phase pattern of samples with *x* = 0, 0.05, and 0.1 (g–i) Out‐of‐plane PFM amplitude image overlaid by phase (red and transparent for both phases). (j–l) Histogram of the measured piezo response with Gaussian function fits around the distinct maxima; the central near‐zero values corresponding to the domain boundary are removed according to the phase data.

Having established the enhanced ferroelectric transition temperature, we now probe the nanoscale polarization states using Piezoresponse Force Microscopy (PFM). PFM measures the out‐of‐plane surface displacement *ΔL* under an applied voltage *ΔV*, from which an effective longitudinal piezoelectric coefficient is derived as *d = ΔL/ΔV*. In mechanically exfoliated crystal flakes, this response primarily reflects the *d_33_* component, as both electric field and displacement align along the z‐axis. However, quantitative extraction of full piezoelectric tensor components remains challenging, since PFM only detects surface displacement rather than 3D deformation, and signals are influenced by local strain, tip‐field distribution, and clamping effects [23]. CIPS exhibits homogeneous out‐of‐plane piezoelectric amplitude with well‐defined ferroelectric domain structures (Figure 4a). In contrast, *x* = 0.05 and 0.1 Li‐doped samples demonstrate pronouncedly reduced out‐of‐plane piezoelectric amplitude with inhomogeneity as shown in Figure 4b,c. For the region with weakened amplitude, unambiguous 180° phase contrast excludes the paraelectric phase such as in In_4/3_P_2_S_6_ (IPS). Interestingly, *x* = 0.05 and 0.1 samples show two typical amplitudes ∼ 4 and ∼15 pm for both in‐phase and antiphase regions, respectively, as shown in Figure 4b,c. In Figure 4g–i, the out‐of‐plane phase pattern is overlaid onto the amplitude pattern. It is evident that each phase correlates with both high and low amplitude regions, confirming that the two amplitude levels are not artifacts arising from asymmetry between opposite polarizations, such as asymmetric tip‐biased electric field and mechanic contact, work function difference at the interface and electrodes. By multiplying with the cos θ of the phase, one can incorporate phase signals into the amplitude to visualize vectorial characteristics of the piezoelectric response. The histograms exhibit the overall statistical results in Figure 4j–l. Note that the pixels in the vicinity of the domain boundary with uncertain phase are excluded, which results in the gap around center *d* = 0 in Figure 4j–l. The CIPS piezoelectric responses reveal a single typical amplitude with both phases, in contrast with two typical amplitudes observed in Li‐doped crystals both for *x* = 0.05 and 0.1. Our results manifest that HP and LP states coexist in the Li‐doped CIPS. In ideally crystallized CIPS, HP phase is metastable and reported only when an additional field is applied, either in an electrically poled system [13, 41, 42], or a heterstructure system, such as CIPS‐IPS system in which an in‐plane lattice mismatch between CIPS and IPS is evident [23], or external pressure [25]. Our work highlights that Li doping provides an effective strategy for stabilizing the HP state.

Besides the piezoresponse, the Cu occupation in the high‐polarization (HP) state is further supported by STEM results. In Figure 2e, the line intensity along the quadruple well passing through Cu^+^ has been evaluated for CIPS and *x* = 0.1 Li doped CIPS. We note that Li‐doped CIPS show a broadened Cu peak width compared with pristine CIPS. The full width at half maximum (FWHM) of Cu peak in CIPS and *x* = 0.1 doped CIPS are 0.124 and 0.190 nm, respectively. As a reference, and to preclude a resolution effect from the STEM, we also evaluated the peak width for In atom along the same direction (Figure 2f). Both CIPS and Li‐doped CIPS show very close peak widths (0.117 vs. 0.132 nm), confirming the peak broadening for Cu atom is not an artifact caused by the image quality. Indeed, the similar resolution for both pictures is also evidenced within the layer, as shown in Figure 2c,d. Therefore, the broadening of the peak along the out‐of‐plane direction implies the occupation of Cu^+^ on both LP and HP states in Li‐doped CIPS, in comparison with the single occupation of LP state in CIPS.

**FIGURE 5:**
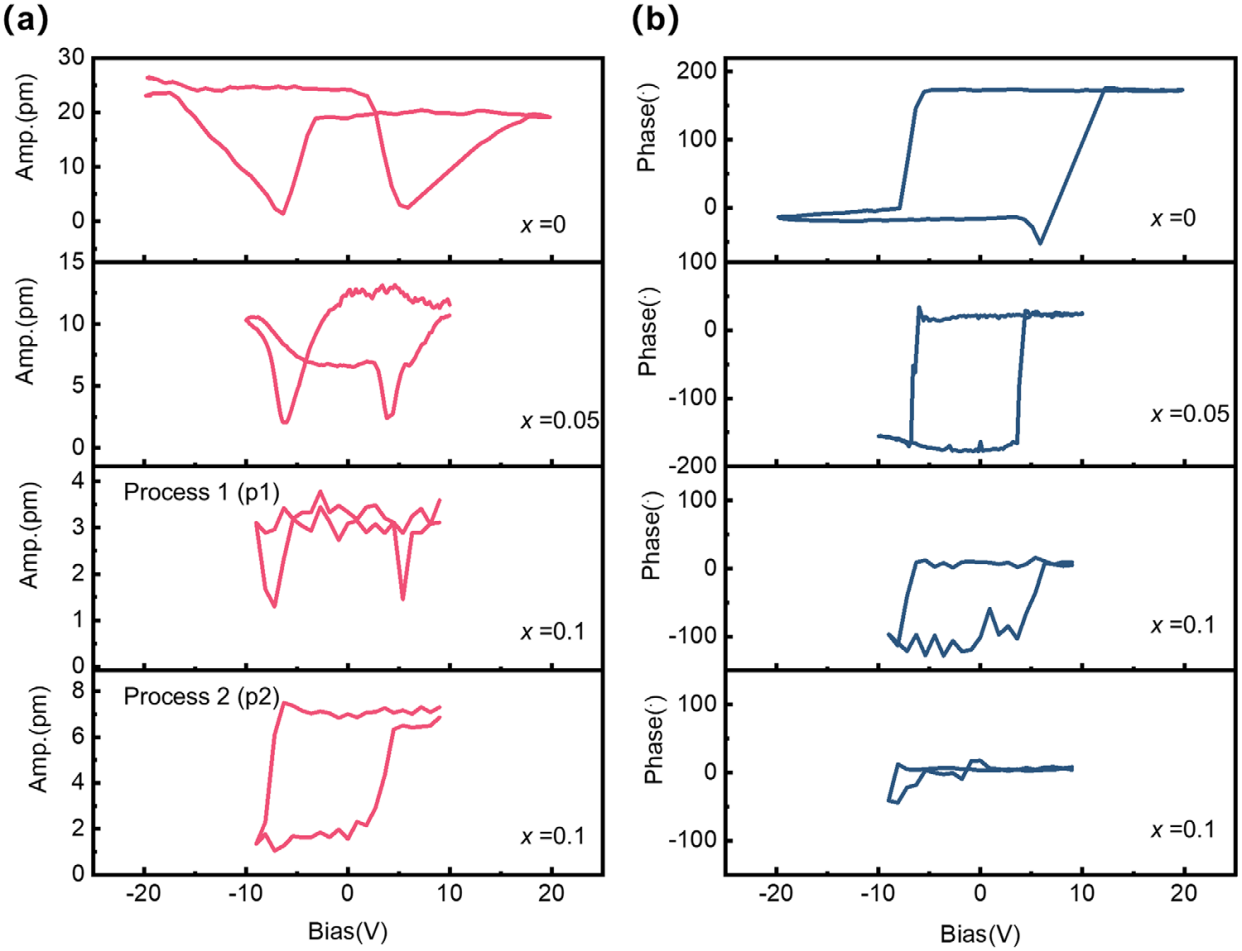
Switching processes of CIPS and Li‐doped samples. PFM piezoelectric response spectrum: (a) amplitude, and (b) phase, during the dynamical polarization switching process of CIPS, *x* = 0.05, and *x* = 0.1 samples.

The switching process between polarization states is also investigated. For CIPS, several various switching processes between distinct polar states have been demonstrated by both PFM piezoelectric response spectrum and *I*–*V* measurements in the cycling voltage sweeping process [13, 23, 26]. Multiple polar states can be achieved as evidenced by the switching of the PFM amplitude. The typical switching process has been reported including +LP→‐LP states. Specially, an ‐LP→+HP process can also occur. The latter process corresponds to a displacement across the vdW gap, which leads to a polarization switch opposite to the external field. In this work, we also conducted a similar investigation on the switching process of Li‐doped samples. Various switching processes are observed as shown in Figure 5. For *x* = 0, two minima in the amplitude and phase reversal are observed, corresponding to the switching between +LP and ‐LP states. Especially, *x* = 0.05 experiences a similar switching process, with the amplitude smaller than *x* = 0 sample, implying a HP state prone to being accessed. More interestingly, for *x* = 0.1 sample, two minima in amplitude correspond to two switching processes (p1 and p2) as shown in Figure 5, with both phases are switched, and the amplitudes are quite small. This manifests a polarization switching between +HP and ‐HP, corresponding to a displacement across the vdW gap. Moreover, the p2 process observed in Figure 5 for *x* = 0.1 sample shows a change of amplitude from ∼6 to ∼2 pm, accompanied by almost no phase switching. Based on the former analysis, this should correspond to a +HP→‐LP process as observed in the CIPS previously [23]. It seems that the most common switching process in CIPS, +LP ⇌ ‐LP, becomes harder to achieve, while switch processes involving HP states are accessible, which also ascribed the stabilized HP state and lowered barrier across the gap induced by Li doping. Many works have manifested polarization switching process across the vdW gap with external voltage, even under an open circuit condition [26]. Our work implies Li doping could be an effective way to lower the switching voltage from tens of volts to a few volts.

Generally, we should note that with doping of Li, the polarization switching process is hindered by the ionic migration process, manifesting as the difficulty in achieving the ideal *P*–*E* loop due to a leaking ionic current and damage to the topography, evidencing a competition between ferroelectric and ionic conductivity. Around this point, some strategies have been developed to control the polarization and ionic conductivity separately, such as by tuning pulse height and duration [14], switching under open circuit rather than closed circuit [26]. This enables an independent manipulation of polarization and ionic conductivity.

### 2.3 Emergence of Topological Polar Textures in Li‐Doped CIPS

**FIGURE 6:**
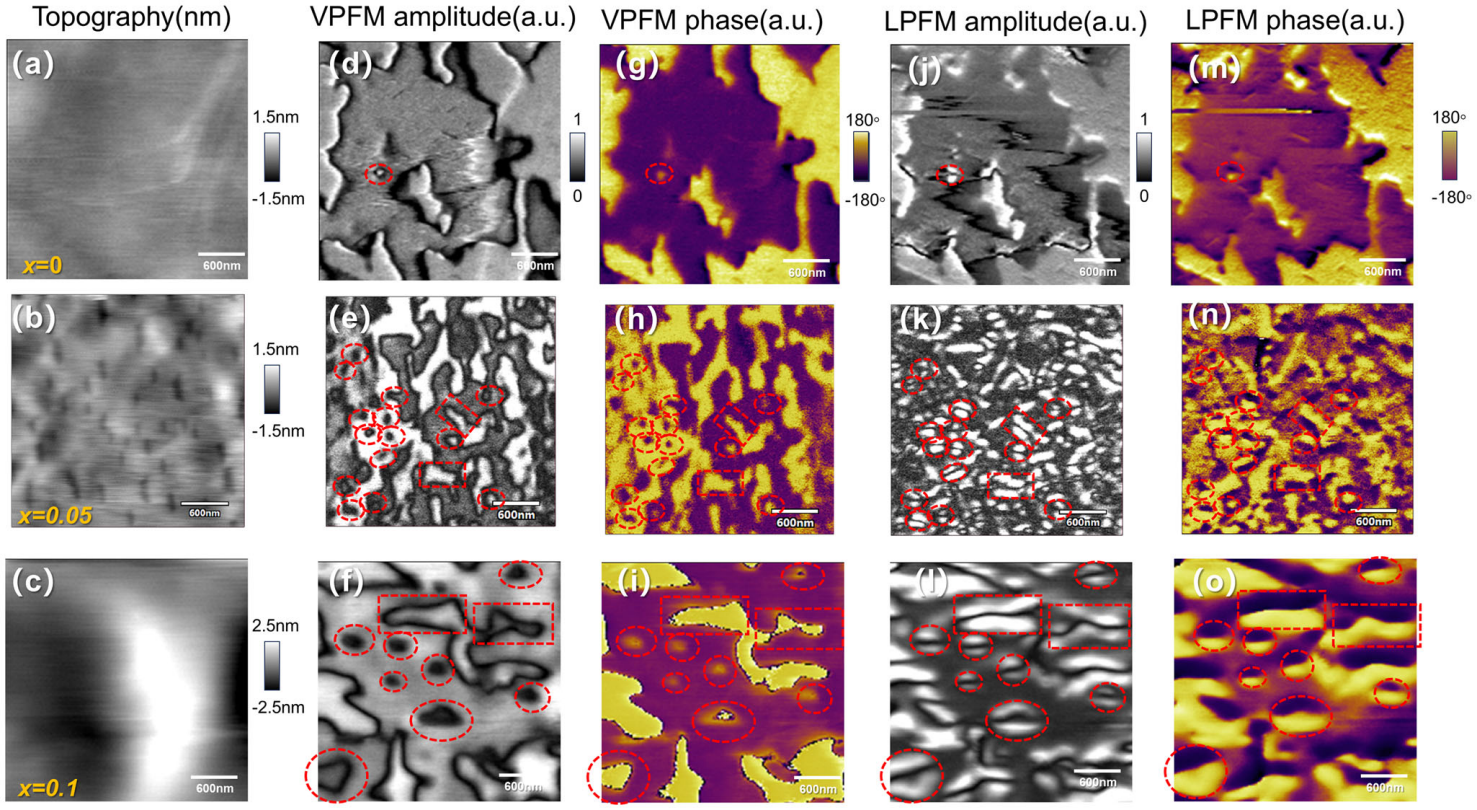
VPFM and LPFM patterns of polar textures in Li‐doped CIPS. (a–c) the topography (d–f) the VPFM amplitudes (g–i) VPFM phases (j–l) LPFM amplitudes and (m–o) LPFM phases, for x = 0, 0.05, and 0.1 Li‐doped CIPS samples, respectively. The red circle featured the coffee‐bean‐like nanodomain, and the red rectangle marked out the labyrinth nanodomain.

Building on the established role of Li‐doping in stabilizing the coexistence of HP and LP states, we embarked on a nanoscale search for emergent topological phenomena using the vector PFM technique. Employing a combined vertical and lateral PFM (VPFM and LPFM) approach, we uncovered a landscape of complex polar textures in *x* = 0.05 and 0.1 Li‐doped samples that is entirely distinct from the pristine counterpart, as shown in Figure 6a–o. To facilitate comparison across datasets, the piezoresponse amplitude variation is normalized to the range of 0 to 1. Critically, the in‐plane piezoelectric responses for *x* = 0.05 and 0.1 Li‐doped samples are not merely a corollary of the vertical polarization, confirming the emergence of a distinct in‐plane polar component. Both in‐plane and vertical components form key ingredients for topological structures. In contrast, the pristine CIPS shows very similar LPFM and VPFM patterns in both amplitude and phase as shown in Figure 6d,g,j,m, implying that the LPFM signal is highly likely due to crosstalk from the VPFM channel and CIPS lacks an in‐plane polarization component. The most compelling evidence emerged from the LPFM amplitude images, which unveiled a high density of characteristic coffee‐bean‐like nanodomain structures in the 0.05 and 0.1 Li‐doped samples as shown in Figure 6k,l. Meanwhile, very few coffee‐bean‐like structures were found in pristine CIPS (Figure 6j). In each coffee‐bean structure, the characteristic feature is defined by dual bright amplitude lobes separated by a central dark node, accompanied by a phase reversal in LPFM, representing the hallmark of in‐plane polarization vortices. The corresponding VPFM signal in the same region shows an isolated particle surrounded by the opposite phase. The area of the VPFM particle is slightly smaller than the coffee‐bean structure, ideally consistent with the core of a polar bubble.

**FIGURE 7:**
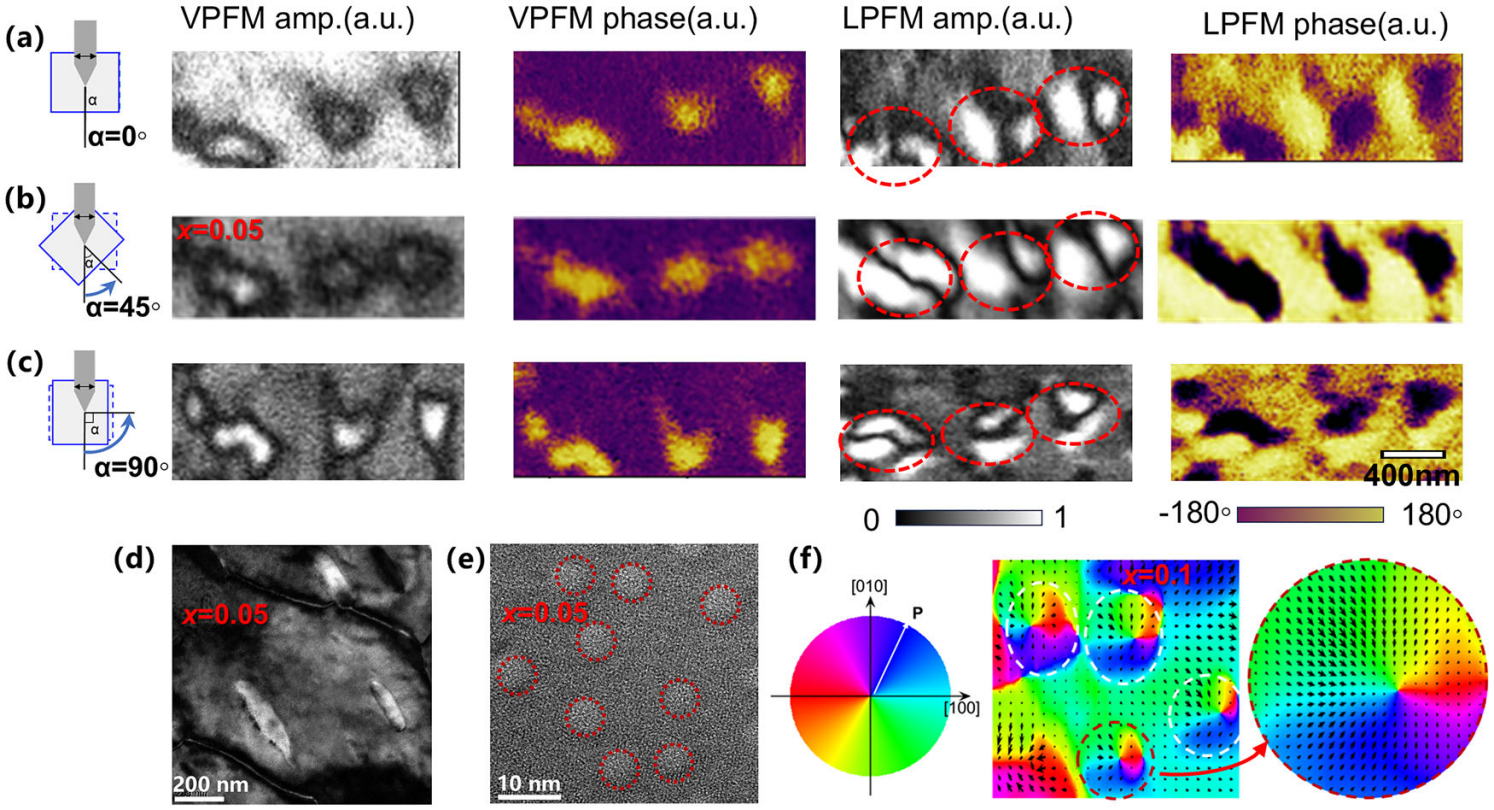
Mapping of in‐plane polar texture by rotating the sample with respect to the cantilever. (a–c) The cantilever‐angle dependent PFM mappings for α  =  0°, 45°, and 90° for x = 0.05 sample. The red circle denotes the bubbles. (d) Dark Field image showing a similar shape and scale with PFM results. (e) High‐Resolution Transmission Electron Microscopy (HRTEM) image showing the inhomogeneity at the nanometer scale, as shown by the dashed line. (f) Polarization vector mapping constructed by the LPFM responses for *x* = 0.1 sample in Figure 6i.

To unambiguously verify their topological nature, we performed vector PFM mapping by rotating the sample relative to the cantilever with α  =  0°, 45°, and 90° as shown in Figure 7a–c, where *α* is the angle between the cantilever and sample. The coffee‐bean morphology shows remarkable rotational invariance. The orange‐and‐purple phase pattern in the LPFM also consistently rotates, providing definitive evidence for a continuous rotation of the in‐plane polarization vector. This rotation symmetry, combined with the orthogonal out‐of‐plane polarization core, allows us to conclusively identify these entities as Néel‐type polar bubbles [7, 27], exhibiting both convergent and divergent core polarizations. The reconstructed in‐plane polarization of *x* = 0.1 sample is shown in Figure 7f.

Strikingly, the Li‐doping concentration acts as a powerful knob to tune the topology of the polar landscape. In *x* = 0.05 Li‐doped CIPS, we observe a high‐density “topological soup” where a multitude of small‐diameter bubbles (∼150 nm) coexist with a fine‐scaled labyrinthine domain structure as shown in Figure 6k, n. Upon increasing the doping to *x* = 0.1, the system undergoes a pronounced coarsening, with the bubble diameter expanding to ∼300 nm and the labyrinth domains widening accordingly. Interestingly, the shared amplitude and phase signatures between the bubbles and the labyrinth domains strongly suggest a common origin. Labyrinth domains are precursors of skyrmions [27]. The fundamental distinction lies in the topological closure: the bubbles represent fully isolated islands where the in‐plane polarization curls around a reversed out‐of‐plane core, while the labyrinth domains lack full encirclement, which prevents this topological charge confinement. The recent work has disclosed the labyrinth domains could be transformed into bubbles in a mechanical manner via the strain and strain gradient [27]. The prior theoretical work investigated the phase diagram and domain structure evolution in spherical nanoparticles of CuInP_2_S_6_ employing the Landau‐Ginzburg‐Devonshire (LGD) approach [43]. It demonstrates the gradient of polarization plays a key role in the formation of labyrinth domains. With a reduced gradient coefficient g44, the density of labyrinth domains is greatly enhanced. According to this argument, the difference in domain appearance for x = 0.05 and x = 0.1 is closely related to the different polarization gradient between LP and HP states. The domain wall in x = 0.05 sample has a lower energy than that of x = 0.1.

Now we turn to discussing the mechanism of emergent topological polar structure in Li‐doped CIPS. In the previous works, the topological textures are typically produced in the heterostructure or multilayer system, which usually includes multiple interactions among strain/strain gradient energy, depolarization energy, and interface interactions [44, 45]. To produce a bubble texture, CIPS as a single‐phase system must contain the inhomogeneity in nanometer scale. In the present work, the inhomogeneity is evidenced by the coexistence of HP and LP states, introduced by the Li‐doping. In Figure 7d,e, we show TEM images for *x* = 0.05 sample at different scales. We see the local microstructure with a size similar to that of the observed polar bubbles. A similar structure was also reported in a previous work [7]. This analogous microstructure is also found in our PFM topography as shown in Figure 6b. Interestingly, one can see that the bubble morphology does not follow the topography pattern. Furthermore, Figure 7e exhibits a rich particle‐like inhomogeneous region on a ∼5nm scale. These inhomogeneities at multiple scales induce rich strain/strain gradient and polarization variations, and provide the strain and strain gradients at the boundary of HP and LP states to favor the emergence of polar bubble, as reported in ref. [27]. This point is also verified in our observation that from Figure 6e,f, where the coffee‐bean regions correspond to a weaker VPFM amplitude than the surroundings, demonstrating HP island is surrounded by LP matrix structure.

While the previous demonstrations of bubbles in CIPS all rely on Cu distribution inhomogeneity or deficiency, it is rarely reported that the topological polar texture could be induced in the perfect CIPS, even under the application of a force or strain gradient. Our approach harnesses a predictable chemical intervention. The incorporation of Li⁺ ions, with their innate nanoscale inhomogeneity, deterministically seeds a widespread and stable HP‐LP phase‐coexistence state. This creates a pervasive energy landscape ripe for the spontaneous formation of strain gradients at the HP‐LP interfaces, which in turn drive the necessary lateral polarization displacements.

Furthermore, we propose that the final topological outcome, whether a closed bubble or an open labyrinth, is dictated by the local geometry of the HP/LP distribution: complete isolation fosters a bubble, while connectivity leads to labyrinths. Given that this nanoscale phase separation is influenced by tunable parameters like doping concentration and thermal history (cooling rate, etc.), our strategy opens the door to the rational engineering of topological phases, promising the flexible fabrication of polar textures with various densities and sizes for future topotronic devices.

### 2.4 Intriguing Flexoelectric Effect in the Li‐Doped CIPS

**FIGURE 8:**
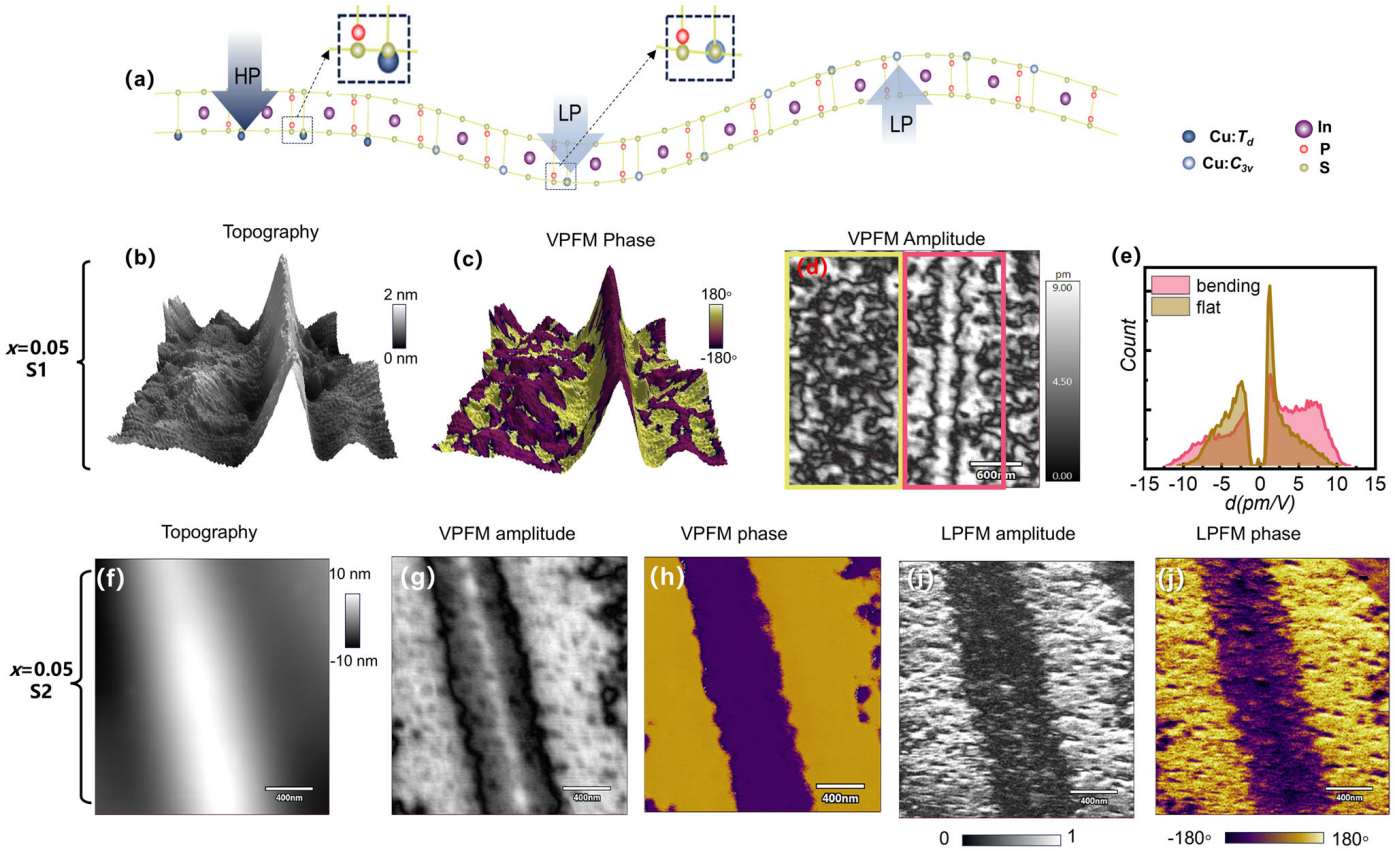
PFM domain pattern associated with topography variation of *x* = 0, 0.05 sample. (a) Schematic view of polarized states of Wrinkles; (b–e) is for *x* = 0.05 sample region S1. (b) 3D AFM topography; (c) VPFM phase imposed on the topography (d) VPFM amplitude mapping, (e) Histogram of the measured piezo response of yellow and pink boxes in (d). (f–i) is for *x* = 0.05 sample region S2. (f) the topography, (g) VPFM amplitude, (h) VPFM phase, (i) LPFM amplitude, and (j) LPFM phase mappings.

In addition to the coexistence of polarization states in Li‐doped CIPS, an intriguing flexoelectric effect is also observed in the *x* = 0.05 Li‐doped sample. A representative domain pattern exhibiting a strong correlation with surface topography is presented in Figure 8. The study focused on a mechanically exfoliated *x* = 0.05 Li doped flake (Samples S1 and S2) that serendipitously contained a microscale wrinkle introduced during the transfer process (Figure 8b,f). On the S1 flake, the VPFM response is homogeneously distributed on the flat part. However, the domain pattern on the wrinkled part is significantly different from the flat regions, where the VPFM phase pattern shows strong consistency with topography (Figure 8c). This direct correlation between polarization orientation and topography suggests that the strain gradient plays a deterministic role in the domain pattern, which is a characteristic signature of the flexoelectric effect [46]. The underlying mechanism, schematized in Figure 8a, originates from the strain gradient across the wrinkle. For upward bending parts in the center of the wrinkle, the upper part of the flake experiences an elongation while the lower part experiences a compression. This strain gradient drives Cu^+^ ions to favor upward polarization, with Cu^+^ displaced toward the expanded space, as schematically shown in Figure 8a. Conversely, the surrounding downward‐bending parts exhibit an opposite phase (Figure 8c). All these observations align well with those reported for CIPS before [46]. However, for this Li‐doped CIPS, we observed an additional critical distinction amplitude variation associated with topography. For CIPS, the strain gradient only switches the phase via curvature, while leaving the amplitude unchanged, which means only +/‐LP states are involved in the switching process. In stark contrast, for *x* = 0.05 Li‐doped sample, a distinctly enhanced amplitude was observed in the bending part compared to the flat part, as shown in Figure 8d. To visualize the variation caused by bending, we analyzed the amplitudes in Figure 8e. The bending region corresponds to an increased area of high piezo amplitude and a reduced low amplitude area, signifying that HP state is forced back into LP state by the strain gradient. Compared with the usual flexoelectric effect, this additional change from HP back to LP state accompanied by a large polarization change (∼10 to ∼4 µC/cm^2^) is promising. This would further enhance the flexoelectric response compared to CIPS. Given that the polarization can be mechanically switched from LP to HP state [25, 31], the strain gradient realizes a reverse process from HP to LP state. These complementary processes form a complete manipulation cycle of polarization states. This opens avenues for the mechanical manipulation of polarization states and applications in electromechanical transduction and sensing devices. We note that this intriguing polarization tuning process between HP and LP states has been elaborated by both experiment and LGD calculation [47, 48]. It was observed that the polarization profile follows the topography in a curved CIPS thin film [47]. According to the LGD calculation, it is shown that the bending curvature can not only switch the sign of polarization, with the polarization direction closely following the bending direction, but also can change between HP and LP phases. Especially, under bending, above a critical bending curvature, HP state can be forced into the LP state, which is fully consistent with our observation here [48].

The evolution of polar textures under controlled strain gradients presents a crucial aspect of their functional manipulation. The recent work has disclosed that a strain gradient applied by AFM tip force can convert the labyrinth domains into polar bubbles at the boundary between HP and LP domains. Here, we investigated both the vertical and in‐plane PFM response on a second wrinkle in *x* = 0.05 Li‐doped Sample S2, as shown in Figure 8f–j. The topography is shown in Figure 8f, with the height of the winkle up to 20 nm. VPFM pattern in Figure 8g,h exhibits a consistent flexoelectric response with S1 in Figure 8b–d. Moreover, the in‐plane PFM response revealed a remarkable variation as shown in Figure 8i,j. At the downward bending region with moderate curvature, the labyrinth domains are replaced by a high density of polar bubbles. This demonstrates a strain gradient can induce the transformation of labyrinth domains into bubbles, as reported in the previous work [27]. However, with further increased gradient in the central upward bending region, the in‐plane amplitude of LPFM is substantially diminished as shown in Figure 8j, implying that the polarization bubble is suppressed, although some bubbles survive. This could be ascribed to the suppressed HP phase in the highly bending part, supporting the argument that the polarization bubble prefers to appear at the boundary between HP and LP states. The predefined strong uniaxial anisotropy in the wrinkle removes the rotational symmetry. This observation highlights a strategy to control the formation and confine a polar texture.

### 2.5 DFT Calculation: Microscopic Mechanism of Li‐Doping Modulated Ferroelectricity and Polar Texture

**FIGURE 9:**
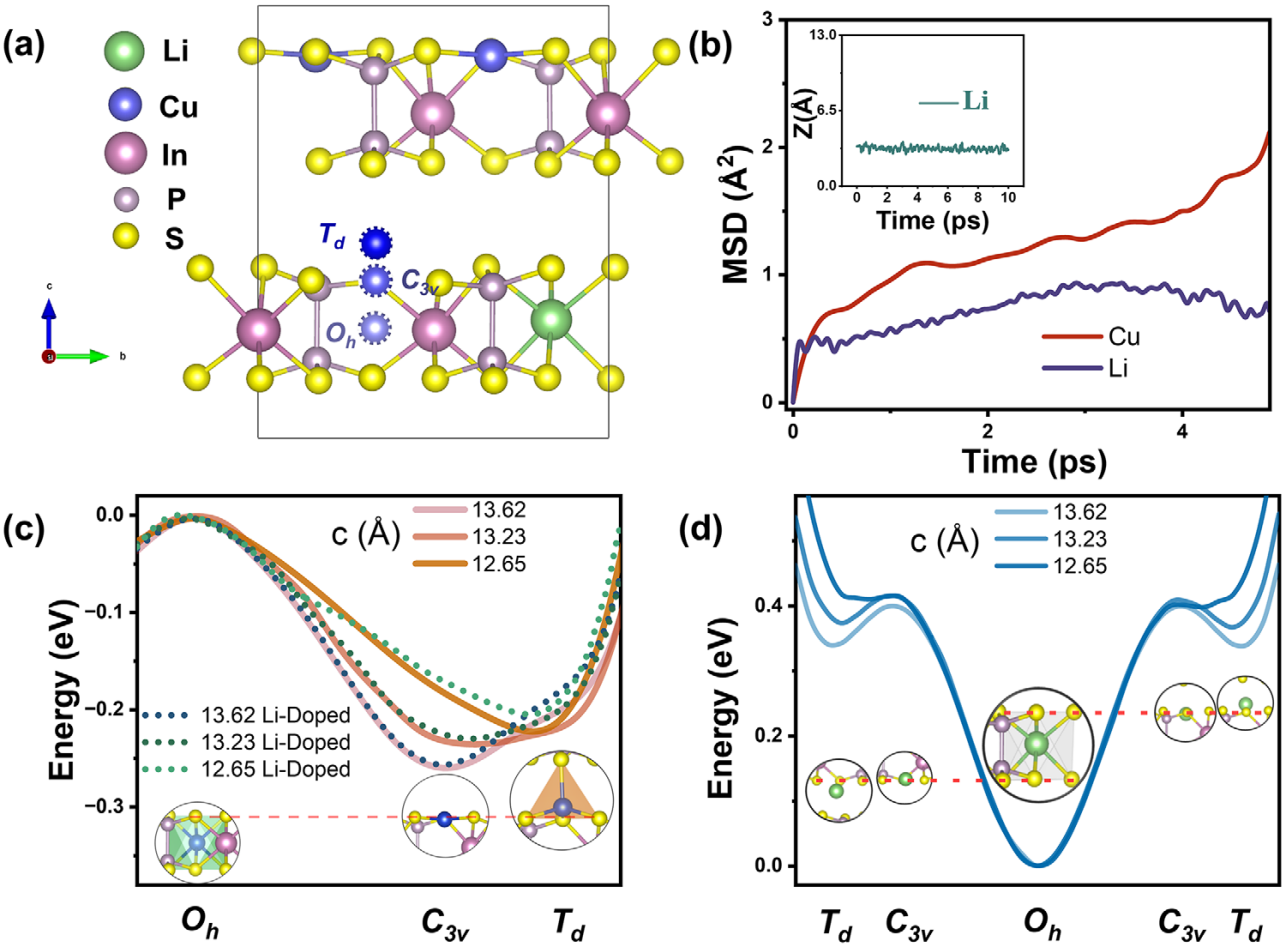
DFT simulation of Li‐doped CIPS. (a) The crystal structure of the Li‐doped CIPS, with Cu^+^ locates at positions corresponding to various polarization state (*O_h_*: paraelectric, *C_3v_*: LP state, *T_d_*: HP state). (b) The root mean square displacement (MSD) for Cu^+^ and Li^+^ as a function of time calculated by AIMD. The inset shows the *z*‐coordinate of Li atom as a function of time during AIMD simulation. (c) The energy profiles of Cu^+^ at varying displacement from *O_h_* site for various lattice parameters *c*. (d) The energy profile of Li^+^ at varying displacement from *O_h_* site. For (c) and (d), the energy at O_h_ site is chosen as a reference.

It is shown that Li doping presented here paves the way for precise property engineering of CIPS. To understand the microscopic mechanisms underlying this variation, a DFT calculation was performed adopting a similar procedure as in ref. [23]. After validating our method against stoichiometric CIPS, we constructed a Li‐doped model (Cu_0_._75_Li_0_._25_InP_2_S_6_, termed Li_0_._25_‐CIPS) by replacing one of four Cu⁺ ions in the unit cell with Li⁺ (Figure 9a). Structural relaxations were carried out using the VASP code with the PBE functional and BJ‐D3 van der Waals correction. The resulting lattice constants (CIPS: *a* = 6.068 Å, *b* = 10.515 Å, *c* = 13.524 Å, *β* = 107.359°; Li‐doped CIPS: *a* = 6.064 Å, *b* = 10.511 Å, *c* = 13.515 Å, *β* = 107.403°) are in close agreement with our experimental results. Given that interlayer spacing significantly affects the polarization states of CIPS, we have performed simulations at various lattice parameters with *c* = 13.62, 13.23, and 12.65 Å for CIPS and Li‐doped CIPS by a compressive strain along the *c*‐axis.

As illustrated in Figure 9a, markers indicate the different positions *O_h_*, *C_3v_*, and *T_d_* for Cu^+^.  We first mapped the energy profile for Cu⁺ displacement from the paraelectric *O_h_* site to the high‐polarization *T_d_* site in both pristine and Li‐doped CIPS, following the established pathway [23]. In this calculation, the Cu *z*‐coordinate was fixed relative to the P─P bond center at each step, while all other atoms were fully relaxed. For pristine CIPS, our results (Figure 9c) agree with previous reports [23]: as the interlayer spacing (c‐lattice parameter) decreases from 13.62 to 12.65 Å, the energy minimum shifts from the *C_3v_* (LP) to the *T_d_* (HP) state. For Li‐doped CIPS, we first determined the stable site for Li⁺. In contrast to Cu⁺, Li⁺ preferentially occupies the central *O_h_* site, with an energy ∼0.4 eV lower than the *C_3v_* site (Figure 9d), consistent with the paraelectric nature of LiInP_2_S_6_ due to the absence of Cu‐3*d* orbitals [24]. With Li⁺ located at the *O_h_* site, the energy profile for the remaining Cu⁺ ions is nearly identical to that in pristine CIPS (Figure 9c). This confirms that low‐concentration Li doping preserves the ferroelectric ground state, aligning with our experiments. Crucially, the reduced interlayer spacing induced by Li doping brings the *C_3v_* and *T_d_* states energetically closer. With the reduction of the gap spacing, the HP (*T_d_* ) state becomes the ground state in Li‐doped CIPS, directly explaining the experimental stabilization of the HP phase. Considering the microscopic inhomogeneity in nanometer scale, a coexistence of HP and LP phases is consistently established and provides the ground for the rich polar textures.

Moreover, the large energy barrier (∼0.4 eV) confining Li⁺ to the *O_h_* site also implies its negligible contribution to ionic conduction. This is confirmed by ab initio molecular dynamics (AIMD) simulations (Figure 9b), which show minimal displacement for Li⁺ compared to the significant migration of Cu⁺ over 5 ps at 300 K. Instead, the enhanced Cu⁺ conductivity stems from the stabilization of the *T_d_* state. Basically, Cu⁺ migration process involves two steps: first, Cu^+^ moves from LP to HP position in same octahedron, and then, Cu^+^ moves to the next octahedron. Therefore, a stabilized HP state lowers the overall energy barrier, reducing the activation energy from ∼0.6 to ∼0.4 eV, as measured experimentally. The preference of Li⁺ on the *O_h_* site is also detrimental to FE order. Therefore, to maintain the FE order, the amount of Lihas to be limited in a small volume. This is consistent with the experimental observation that *x* = 0.13 sample has no long‐range ferroelectric order (see Figure 3d).

## 3 Conclusion

In summary, we demonstrate that minimal Li⁺ doping (*x* ≤0.1) in van der Waals CIPS provides a potent chemical strategy to co‐enhance ferroelectricity and generate rich polar textures, such as bubbles and in‐plane labyrinth domains. The smaller ionic radius of Li⁺ reduces the interlayer spacing, which strengthens the interlayer Cu─S bonding. This mechanism underlies all key observations: an increase in Curie temperature to 327 K, the stabilization and coexistence of low‐ and high‐polarization (LP/HP) states, and a reduction in the activation energy for Cu⁺ migration from ∼0.6 to ∼0.4 eV. Interestingly, rich polar bubbles and labyrinth domains emerge directly from the HP and LP boundary in Li‐doped samples, benefiting from the strain/strain gradient and polarization variation caused by Li doping. Furthermore, an unusual flexoelectric effect, in which strain gradients can reversibly switch the HP state back to the LP state is found. This effect provides a mechanical means to manipulate the polar bubble using strain gradients. As an effective and facile strategy, Li‐doping can finely tune the ferroelectricity, polar textures, and ionic conductivity, thereby paving the way for advanced electronics, neuromorphic devices, and topotronics.

## 4 Experimental Section

### 4.1 Sample Preparation

Synthesis of Cu_1‐x_Li_x_InP_2_S_6_ Single Crystals. Our CuInP_2_S_6_ crystals were grown by chemical vapor transport in evacuated silica ampules. The stoichiometric ratio of the raw materials Cu, In, P, and S was mixed as precursors. Iodine was used as the transport agent in the reaction process. These powders were ground together in a mortar and pestle inside a nitrogen‐filled glovebox and subsequently loaded into an evacuated quartz ampule. The temperature of the evaporation and crystallization zones was set to 750 °C and 650 °C, respectively, and the chemical reaction was carried out for 2 weeks. After cooling, the ampules were opened, and the crystals were extracted. The crystals were platelets ∼100 mm^2^ or smaller in area and <1 mm thick. The crystals presented in Figure 1a are among the crystals we obtained. Thin CIPS flakes were extracted by Scotch‐tape‐based exfoliation and transferred onto conductive Au/SiO_2_/Si substrates.

### 4.2 Raman Measurement

Temperature‐dependent Raman spectra were collected using a confocal Raman system (WITec). The incident excitation (532 nm) was directed onto the sample CIPS and Cu_1‐x_Li_x_InP_2_S_6_ crystals mounted within a temperature control station. The laser power was set to a few milliwatts to avoid additional heating from the laser.

### 4.3 PFM Measurement

PFM measurement was carried out on a commercial atomic force microscope (Asylum Research MFP‐3D) under both resonance‐enhanced modes. The in‐plane and out‐of‐plane PFM measurements were done in the LPFM and VPFM modes, respectively. In VPFM mode, a conductive probe with a Pt/Ir coating was driven with an AC voltage (*V_AC_* = 1 V) under the tip‐sample contact resonant frequency (∼340 kHz). In LPFM mode, a conductive probe with a Pt/Ir coating was driven with an AC voltage (*V_AC_* = 0.8 V) under the tip‐sample contact resonant frequency (∼650 kHz).

### 4.4 NMR Measurement

Li nuclear magnetic resonance (NMR) were performed on a Bruker AVANCE NEO 400 mHz NMR spectrometer at 9.4 T (Larmor frequency ω*_0_* = 155.5 mHz) with a 4 mm iprobe HX MAS probe at room temperature, and 343 K. Li chemical shift is referenced to LiCl (1 m a.q.) as 0 ppm.

### 4.5 Other Characterization

The complex dielectric permittivity was measured using a commercial LCR meter (Agilent E4990A) in the frequency range 20 Hz–1 mHz. All temperature‐dependent dielectric measurements have been performed on the warming process with a controlled temperature rate of 5 K/min using a PPMS system. Silver paste has been used for contacting. The morphology image and the EDS mapping results were obtained by thermal field emission environmental EDS (Quanta 400 FEG). XRD analysis was carried out using the X‐ray diffractometer platform (Miniflex 600).

### 4.6 DFT Calculation

The first‐principles calculations were performed via the Vienna Ab initio Simulation Package (VASP) using the generalized gradient approximation (GGA) with the projected augmented wave method (PAW) with a 450 eV plane‐wave energy cutoff. The Perdew‐Burke‐Ernzerhof (PBE) function was adopted for the exchange‐correlation functional [49]. A Gamma‐centered k‐point grid of 9 × 5 × 5 was applied for sampling the Brillouin zone. The energy and force threshold for structural optimization are 10^−5^ eV and 0.01 eV/Å. The DFT‐D3 vdW correction with the Becke‐Jonson damping method was considered [50].

## Author Contributions

Y.Z. and X.L. conceived the research idea and supervised the project. L.G. designed the experiment procedure. L.G. and Z.Z. conducted the sample preparation, structural characterization, and dielectric measurement. L.G. and F.S. carried out the PFM measurements. W.L. carried out the DFT calculations and analyses. Z.S., J.L., and Y. Z. conducted the TEM measurements and data analysis. W.C., X.Z., and X.L. jointly discussed the polarization configuration analysis and contributed to the conclusion. H.J. and W.X. assisted with the device fabrication and data acquisition. L.G. and X.L. drafted the manuscript, and Y.Z. revised it. All authors discussed the results and approved the final version of the manuscript.

## Conflicts of Interest

The authors declare no conflict of interest.

## Supporting information




**Supporting File**: advs73657‐sup‐0001‐SuppMat.docx.

## Data Availability

The data that support the findings of this study are available from the corresponding author upon reasonable request.
