# Supplementary material for: Chemical Doping Engineering of Polarization and Topological Textures in van der Waals Ferroelectric CuInP2S6
Source: Adv Sci (Weinh). 2026 Jan 5;13(16):e23774. doi: 10.1002/advs.202523774 (PMC13042472; doi:10.1002/advs.202523774)
Supplement: Supplementary file 1 — Supporting File: advs73657‐sup‐0001‐SuppMat.docx. [file ADVS-13-e23774-s001.docx]

Supporting Information

**Chemical Doping Engineering of Polarization and Topological Textures in van der Waals Ferroelectric CuInP₂S₆**

*Lei Gao, Wenhao Li, Fei Sun, Ziyi Zhou, Zhihao Shen, Jianwei Liang, He Jiang, Weiming Xiong, Weijin Chen**, Xiaoyue Zhang, Yi Zhang, Xinzhi Liu^*^, Yue Zheng^*^*

L. Gao, W. Li, F. Sun, Z. Zhou, Z. Shen, J. Liang, H. Jiang, X. Zhang, X. Liu, Y. Zhang, and Y. zheng

Guangdong Provincial Key Laboratory of Magnetoelectric Physics and Devices, Centrer for Physical Mechanics and Biophysics, Institute of Neutron Science and Technology, School of Physics, Sun Yat-sen University, Guangzhou 510275, China; State Key Laboratory of Optoelectronic Materials and Technologies, Sun Yat-sen University, Guangzhou 510275, China

W. Chen

Guangdong Provincial Key Laboratory of Magnetoelectric Physics and Devices, Centrer for Physical Mechanics and Biophysics, School of Physics, Sun Yat-sen University, Guangzhou 510275, China; State Key Laboratory of Optoelectronic Materials and Technologies, Sun Yat-sen University, Guangzhou 510275, China; School of Materials, Sun Yat-sen University, 510275, Guangzhou, China

W. Xiong

State Key Laboratory of Optoelectronic Materials and Technologies, Sun Yat-sen University, Guangzhou 510275, China; Center on Nanoenergy Research, Guangxi Key Laboratory for the Relativistic Astrophysics, School of Physical Science & Technology, Guangxi University, Nanning 530004, P. R. China

E-mail: liuxzh39@mail.sysu.edu.cn; zhengy35@mail.sysu.edu.cn


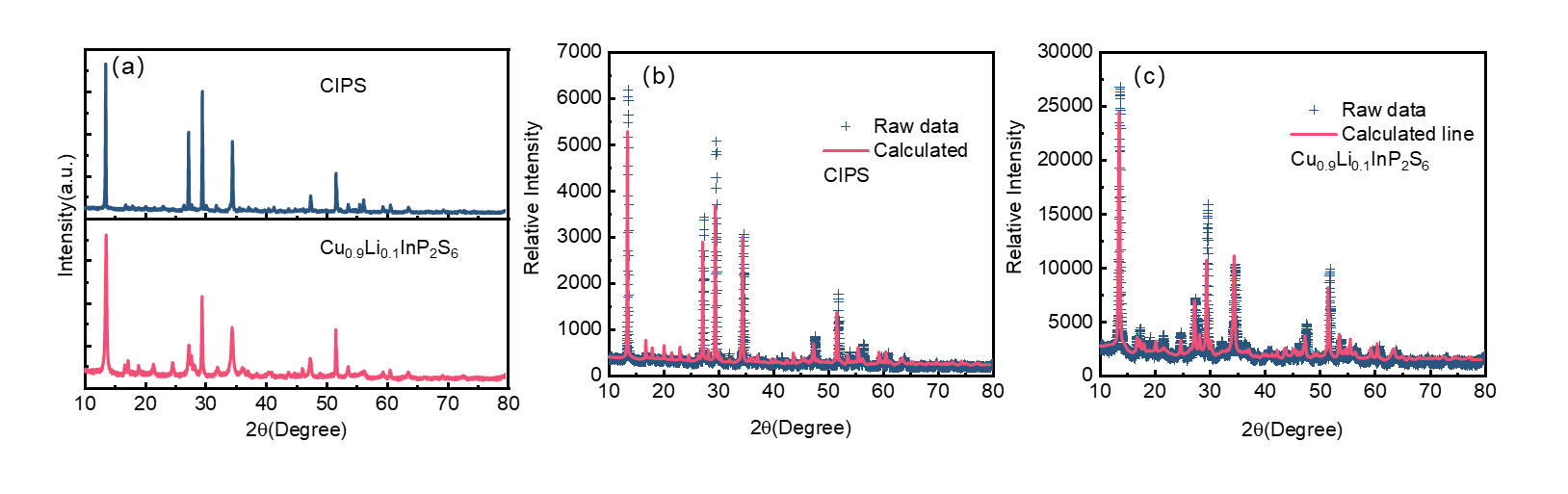


**Figure S1.** (a) XRD patterns of the samples. Rietveld refinement of the powder XRD pattern of CIPS (b) and *x*=0.1 doped CIPS (c).

Figure S1 presents the Rietveld refinement results of the powder X-ray diffraction (XRD) patterns for CIPS and x=0.1 Li-doped CIPS. The experimental data are denoted by dotted lines, and the refined calculated patterns are shown as solid lines, obtained using the Fullprof software. Both compounds were refined based on the *Cc* space group of CIPS. Upon Li doping, the peak positions remain largely unchanged, while with a slight shift and broadening. These results indicate that the introduction of Li preserves the original crystal structure, yet introduces additional disorder. The refined lattice parameters are as follows: CIPS: a=6.102 Ǻ, b=10.574 Ǻ, c=13.204 Ǻ; x=0.1 Li-doped CIPS: a=6.115 Ǻ, b=10.587 Ǻ, c=13.184 Ǻ.

**
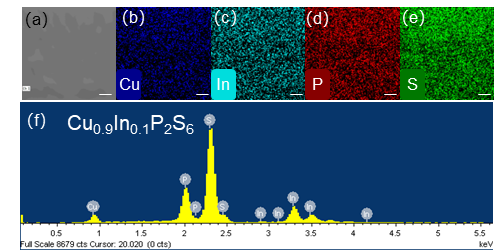
**

**Figure S2.** (a) High-resolution scanning transmission electron microscopy (HR-STEM) for the *x*=0.1 Li-doped CIPS and the corresponding EDS compositional maps for (b) Cu, (c) In, (d) P, and (e) S elements of (a). (f) EDS spectra collected of *x*=0.1 doped CIPS. The scalebar is 20 nm.

Figure S2 provides evidence for elemental distribution of the *x*=0.1 doped CIPS flakes. Elemental mapping performed via high-angle annular dark-field (HAADF) imaging demonstrates Cu, In, P, and S across the sample, with no observable agglomeration or voids. This elemental dispersion further confirms the phase uniformity as well as the high quality of the single crystal. Additionally, the corresponding EDX peaks reflect favorable detection rates for each element, supporting the consistency of the sample composition.

**
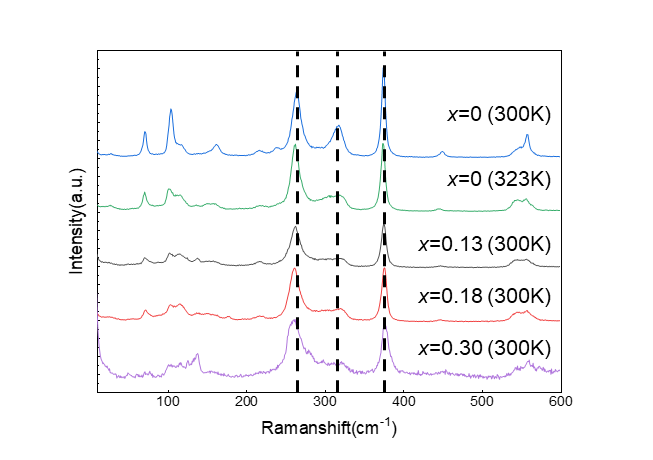
**

**Figure S3** Raman spectrums of the Li-doped samples with various ratios.

Figure S3 compares the Raman spectra of CIPS and x=0.13, 0.18, 0.3 Li-doped CIPS. As the temperature changes from 300 K to 323 K for CIPS, the Cu-associated vibrational mode near 320 cm⁻¹ shows clear phase transition sensitivity. With increasing lithium content up to x = 0.13~0.18, Raman spectrum peaks retain their positions, but the peak of 320 cm⁻¹ exhibits noticeable intensity reduction, reflecting the suppression of ferroelectric order while the host crystal structure remains intact. At a higher doping level of *x* = 0.3, additional Raman peaks emerge around 130 cm^-1^, indicating a structural change or impurity phase occurs. The spectrum variation confirms the disappearance of ferroelectricity under high lithium doping, which is in consistent with the dielectric spectrum shown in Figure 3. Importantly, the retained S-P-S (275 cm^-1^) and P-P (380 cm^-1^) vibration features suggest that the basic PS3 framework-based local structure is partially preserved, likely forming a eutectic-like or composite phase.


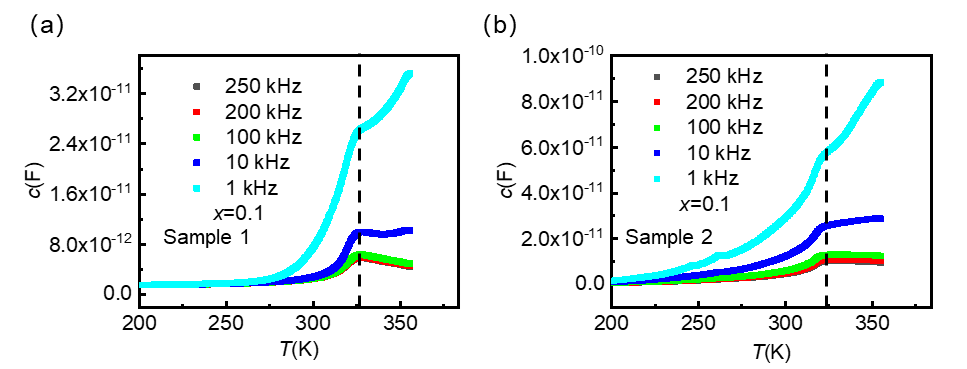


**Figure S4.** The temperature dependence of capacitance (dielectric constant) for different *x*=0.1 Li-doped CIPS samples.

Figure S4 validates the universal enhancement of the ferroelectric-paraelectric transition temperature induced by Li doping. Capacitance measurements performed on two independent x=0.1 Li doped CIPS samples consistently reveal transition peaks near 325 K, confirming the reproducibility of the phase transition behavior. These results provide clear evidence that Li doping effectively elevates the ferroelectric-paraelectric transition temperature.


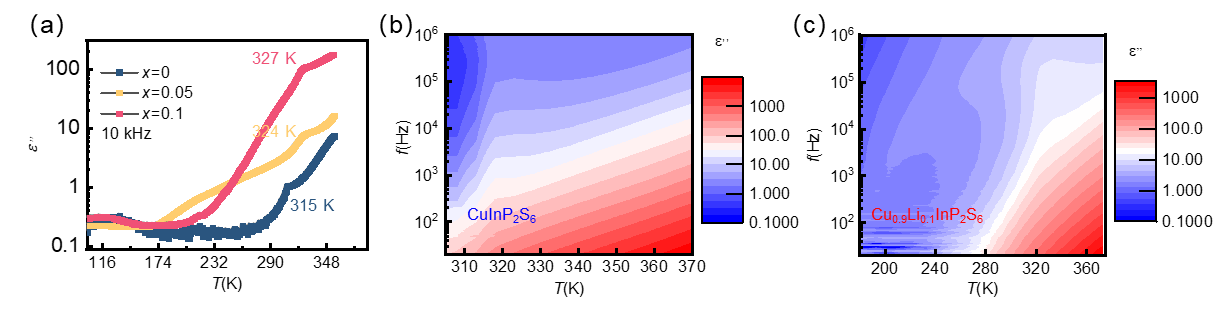


**Figure S5.** (a) The temperature dependence of imaginary part of permittivity $\varepsilon^{''}$ of Cu_1-x_Li_x_InP_2_S_6_ at 10 kHz. (b,c) Contour plots of the dielectric imaginary part of permittivity $\varepsilon^{''}$ for CuInP_2_S_6_ (b) and Cu_1-x_Li_x_InP_2_S_6_ (c).

Figure S5 presents the temperature- and frequency-dependent imaginary part of the dielectric behavior in CIPS and x=0.05, 0.1 Li-doped CIPS. Figure S5 a displays the imaginary part of the dielectric constant ($\varepsilon^{''}$) measured at 10 kHz, from which the ferroelectric–paraelectric transition temperatures are identified as 315 K, 324 K, and 327 K for x=0, 0.05, 0.1 Li-doped samples, respectively. These results are consistent with the real permittivity ($\varepsilon^{'}$) data shown in Figure 3. As further demonstrated in Figure S5 b and c, Li doping leads to a notable increase in the transition temperature, confirming its role in stabilizing the ferroelectric phase. To quantitatively evaluate the influence of Li doping on ion transport characteristics, the activation energy (Eₐ) was calculated using $\sigma=2\pi f\varepsilon_{0}\varepsilon^{''}$, where $\sigma$ denotes the electric conductivity, $f$ is frequency, and $\varepsilon_{0}$ is vacuum permittivity, see the main text.


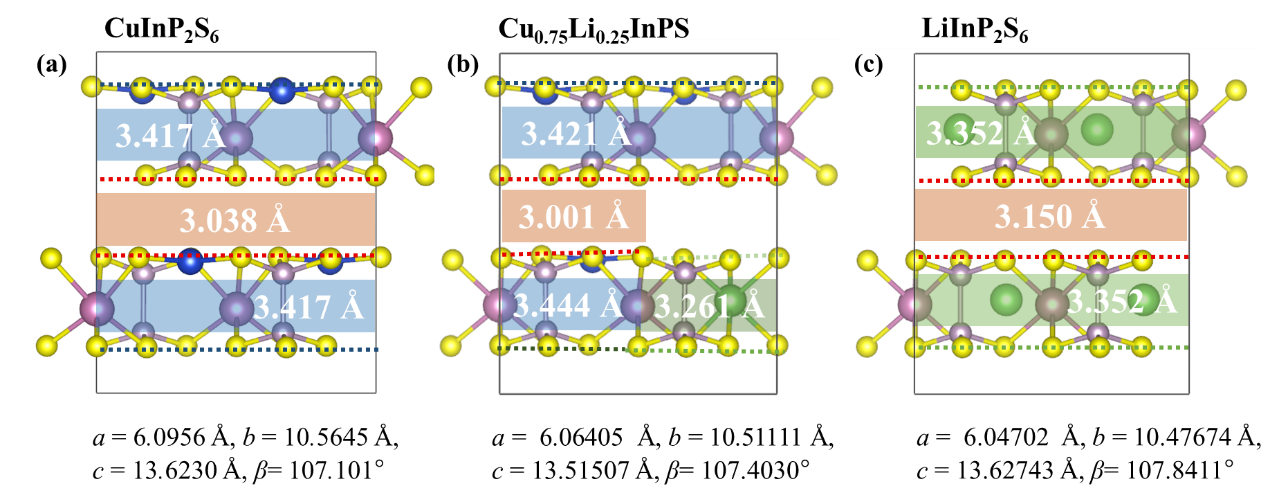


**Figure S6.** Detailed data for different structures, showing the lattice parameters after full relaxation for (a) CIPS, (b) Li-doped CIPS, and (c) LiInP_2_S_6_ (LIPS) structures, along with the thickness of each layer and the magnitude of the VDW gap.
